# Sexual health of Syrian women in protracted forced displacement: the syndemic interplay of violence, war trauma, poor mental health and food insecurity

**DOI:** 10.1136/bmjph-2025-002561

**Published:** 2025-07-05

**Authors:** Sasha Fahme, Ghina R Mumtaz, Maia Sieverding, Sara Chehab, Mostafa El Nakib, Laith Abu-Raddad, Jennifer Downs, Myung Hee Lee, Jocelyn DeJong, Daniel Fitzgerald

**Affiliations:** 1 Center for Global Health, Weill Cornell Medical College, New York, USA; 2 Epidemiology and Population Health Department, American University of Beirut Faculty of Health Sciences, Beirut, Lebanon; 3 Center for Infectious Diseases Research, American University of Beirut Faculty of Medicine, Beirut, Lebanon; 4 Department of Health Promotion and Community Health, American University of Beirut Faculty of Health Sciences, Beirut, Lebanon; 5 National AIDS Control Program, Beirut, Lebanon; 6 Department of Population Health Sciences, Weill Cornell Medical College, New York, USA; 7 Infectious Disease Epidemiology Group, Weill Cornell Medicine, Doha, Qatar; 8 World Health Organization Collaborating Centre for Disease Epidemiology Analytics on HIV/AIDS, Sexually Transmitted Infections, and Viral Hepatitis, Weill Cornell Medicine, Doha, Qatar

**Keywords:** Syndemic, Sexual Health, Sexually Transmitted Diseases, Sociodemographic Factors, Violence

## Abstract

**ABSTRACT:**

**Background:**

Women who have been forcibly displaced in complex humanitarian settings suffer from poor sexual health, though the association with violence and war trauma is poorly characterised. We investigated sexual health outcomes and their relationship to violence and trauma among women living in a protracted forced displacement setting.

**Methods:**

This cross-sectional study was conducted in an urban refugee camp in Beirut, Lebanon. Participants were Syrian women who are refugees aged 18–49 years. Community health workers conducted door-to-door recruitment and administered a survey to assess violence and trauma. Capillary blood and vaginal swabs were collected for sexually transmitted infection (STI) testing. Sexual health outcomes included gynaecologic symptoms, self-reported lifetime STI history and current STIs. The relationship of sexual health outcomes with violence, trauma and other factors was assessed using Bayesian Gaussian copula models.

**Results:**

Recruitment and data collection were conducted from February to September 2023. Of 305 eligible participants, 250 (82.0%) consented for study participation. The mean age was 30.3 (SD±6.9) years. Over 80% of women disclosed experiencing gender-based violence. Participants experienced a median of 12 (IQR 7) traumatic war events; 197 (78.8%) met criteria for post-traumatic stress disorder (PTSD) and 125 (50.0%) had severe food insecurity. Among a subset (n=216), current gynaecologic symptoms (80.1%) and lifetime STI history (63.4%) were common. We detected one case of *Trichomonas vaginalis*. By graphical models, gynaecologic symptoms and STI history were significantly associated with sexual violence, which clustered with physical and emotional violence, war trauma, PTSD and severe food insecurity (posterior edge inclusion probability >0.5).

**Conclusion:**

In Lebanon, the sexual health of Syrian women who are refugees is closely linked to a syndemic of violence, war trauma, poor mental health and food insecurity. A syndemics-informed approach is urgently needed to address the needs of forcibly displaced women.

**WHAT IS ALREADY KNOWN ON THIS TOPIC**

- The sexual health of women who are refugees in protracted forced displacement settings is underprioritised despite a burden of unmet needs.
- In West Asia and North Africa, a region from which over half of the global forcibly displaced population originates and is hosted, roughly one in three ever-partnered women have experienced gender-based violence, but most studies exclude refugees.
- Among displaced Syrian women, who represent the largest forcibly displaced female population globally, the prevalence of gender-based violence and its association with sexual health outcomes are poorly understood.
- A syndemics model relating violence, poverty and sexual health may clarify the relationships between these factors and inform targeted interventions.

**WHAT THIS STUDY ADDS**

- We demonstrate a significantly higher burden of gender-based violence (81.2%), including sexual violence (22.0%), than has been previously described in the literature.
- Bayesian Gaussian copula models illustrate the syndemic clustering of sexual, physical and emotional violence, severe war trauma exposure, post-traumatic stress disorder and severe food insecurity with sexual health outcomes, including gynaecologic symptoms (80.1%) and lifetime sexually transmitted infection (STI) history (63.4%).
- There is a discrepancy between lifetime STI history and current STIs in the context of widespread self-prescribed antimicrobial use among over half of symptomatic participants.

**HOW THIS STUDY MAY AFFECT RESEARCH, PRACTICE OR POLICY**

- This research sheds light on the syndemic interplay of modifiable psychosocial factors, including violence, war trauma and food insecurity, which perpetuate poor sexual and mental health outcomes among a vulnerable refugee population.
- Violence and post-traumatic stress disorder may render women susceptible to reproductive tract infections through immune dysregulation; the unfettered use of self-prescribed antimicrobials may promote vaginal dysbiosis, further exacerbating gynaecologic symptoms and increasing antimicrobial resistance, an under-researched but growing area of concern in conflict and displacement settings.
- A multidisciplinary response adopting the framework for syndemic effects in complex humanitarian emergencies is urgently needed to address social and health disparities in this population.
- Integrating refugee women-led interventions into local health systems may be an effective approach to improving women’s health and achieving health equity in this protracted forced displacement setting.

## INTRODUCTION

 Conflict-affected women who are refugees suffer from poor sexual health.^1 2^ As the rapidly growing global refugee population is becoming increasingly urbanised within low and middle-income countries,^3^ often in a context of protracted displacement,^4^ social and economic vulnerabilities may increase the burden of poor sexual health outcomes among women and girls.^1 2 5^ Yet, women’s sexual health needs remain poorly characterised in protracted forced displacement settings and are therefore not addressed. Challenges in communicable disease surveillance, minimal sexual health services provision and competing health priorities limit capacity to improve sexual healthcare among conflict-affected and forcibly displaced populations.^2 6^

In West Asia and North Africa (WANA), decades of armed conflict and political instability have destabilised health systems as well as caused the forced displacement of millions of women, who may be at disproportionate risk of poor sexual health outcomes.^7 8^ Displaced Syrian women, the largest population of forcibly displaced women globally,^9 10^ endure humanitarian crises characterised by extreme poverty, restricted mobility and dismally low access to healthcare, potentially impacting their sexual health.^110^,^13^ Early marriage, intimate partner violence and transactional sex may be prevalent in this population,^113^,^15^ though most data are qualitative and non-generalisable. Limited cross-sectional sexually transmitted infection (STI) prevalence data are inconsistent and limited to clinic attendees.^16^,^19^ One study in Turkey indicated that trichomoniasis may be as common among displaced Syrian women as among sex workers.^16^ In contrast, studies conducted in Lebanon have found lower prevalence levels of curable STIs among displaced Syrian women, including pregnant women engaged in antenatal care.^17^,^19^ To date, no studies have examined the association between social determinants of health shaped by humanitarian crises and STIs among Syrian women who are refugees.

Our formative, qualitative work for this study demonstrated that forcibly displaced Syrian women experience intersecting forms of systemic and intrapersonal violence, poverty and stigma, which may drive negative sexual health outcomes and impact mental health.^20^ Women’s lived sexual health experiences were characterised by sexual harassment, exploitation, trafficking, intimate partner violence and rape, including in marriages.^20^ Experiences were further complicated by women’s social environments, frequent isolation and extreme poverty.^20^ Challenges with notifying and engaging partners in sexual healthcare, largely due to STI stigma, and limited access to healthcare were major barriers to effective treatment, potentially exacerbating STI recurrence and sequelae.^20^

Taken together, these social, political and economic disparities, and their association with sexual health conditions, suggest the emergence of a potential syndemic, defined as the synergistic occurrence of two or more diseases in the context of population-specific social, political and economic inequities.^21^ The syndemics model has been used to describe the relationship between violence, poverty and sexual health among other refugee populations, including adolescents in Uganda, where HIV vulnerabilities were shown to be mediated by gender-based violence, poverty, substance use and poor mental health.^22 23^ A bidirectional relationship was similarly demonstrated between gender-based violence and infectious disease outcomes, including Zika, Ebola and COVID-19, among humanitarian crisis-affected women and girls in Sub-Saharan Africa.^24^ The authors conclude that syndemic policies which address broader gender inequities and emphasise community resilience are needed in order to mitigate emerging epidemics.^24^

Because the syndemics model addresses upstream social factors, which impact an individual’s experience of a disease, it necessitates that corresponding health interventions be population-centric rather than disease-centric.^25^ This novel approach has unique implications for designing interventions in humanitarian settings. A syndemics-informed response in such contexts is often multidisciplinary and addresses disparities related to political violence, forced displacement and disruption of social services, all of which may impact healthcare-seeking behaviours and access to care among refugee and other marginalised populations.^26^ Still, while the syndemics model has been applied in other complex humanitarian contexts, it remains underutilised among Syrian women who are refugees, despite the confluence of disparities which compromises their sexual health. To date, the relationship between sexual health outcomes and violence and trauma among Syrian women in protracted forced displacement settings has not been systematically studied.

The purpose of this study is to quantify sexual health outcomes, including the prevalence of gynaecologic symptoms, self-reported lifetime STI history and current STIs, and determine their relationship to violence and trauma among a community-based sample of Syrian women living in an urban-protracted forced displacement setting. We hypothesise that gynaecologic symptoms and STIs are common and associated with violence and war trauma among this forcibly displaced population.

## METHODS

### Study design

This cross-sectional study was conducted as part of an exploratory-sequential mixed-methods study and was informed by qualitative in-depth interviews.^20^ The study was initially designed to assess demographic and psychosocial correlates of current STIs. After initial enrolment, data emerged from women on a broader range sexual health issues. Therefore, the study protocol was amended; questions related to sexual health history (online supplemental A) and two additional endpoints (gynaecologic symptoms and STI history) were added to the protocol.

### Study setting

An estimated 1 to 1.5 million Syrian refugees are resettled in Lebanon, representing the highest per capita refugee population globally.^9^ The majority of Syrian refugees in Lebanon are not registered with the United Nations Refugee Agency, which may compound their vulnerability as unregistered refugees often have limited access to healthcare.^10 27^ The study was conducted in the Bourj-al-Barajneh refugee camp in Beirut, Lebanon. Originally established by the Lebanese government in 1949 to house Palestinian refugees,^28^ the Bourj-al-Barajneh refugee camp today is an urban slum spanning one square kilometre in the southern district of the city with overcrowded housing conditions and poor water, sanitation and hygiene infrastructure.^29^ Approximately half of the over 18 000 camp residents are now Syrian refugees, displaced from Syria since 2011.^28^ While nationally representative data among Syrian refugees in Lebanon are limited, those resettled in Beirut, where the refugee camp is located, have been shown to have comparable levels of economic vulnerability and access to healthcare as refugees elsewhere in Lebanon.^10^ Notably, there are several clinics operated by local and international non-governmental organisations (NGOs) offering subsidised primary care services proximate to the refugee camp.

Data were collected from February 2023 to September 2023 at a community centre operated by our partner, Beit Atfal Assumoud, an NGO that has provided primary care, sexual and reproductive health services, psychosocial counselling and educational support within refugee camps in Lebanon for over four decades.

### Study participants and recruitment

Study participants were adult women refugees from Syria, between 18 and 49 years of age, who arrived in Lebanon following the onset of the Syrian conflict in 2011 and maintained residence in the Bourj-al-Barajneh refugee camp for a minimum of 6 months prior to the study. The latter inclusion criterion was to minimise confounding, theorising that newly resettled residents may have had differential sexual health exposures based on previous place of residence. Recently arrived refugees may also have comparatively limited access to local health services, and, therefore, a potentially increased risk of STIs.

Three female community health workers (CHWs), who are refugees residing in the camp, were trained to conduct recruitment and all data collection. We adopted a three-pronged, community-based sampling strategy within the refugee camp that entailed: (1) CHWs advertising flyers with study contact information throughout public areas within the refugee camp; (2) CHWs directly approaching women in marketplaces and other communal spaces to inform them about the study and assess their interest and eligibility; and (3) door-to-door recruitment with CHWs visiting households in areas of the camp known to have a high population of Syrian refugees. The latter was based on the principles of adaptive cluster sampling, which stipulate that similar populations such as refugees tend to live close together, particularly within urban settings.^30^ Thus, CHWs arbitrarily selected an apartment within a building and, if the residents were Syrian refugees, proceeded to sample the remaining households within the same building. All women meeting inclusion criteria within a single household were invited to participate. Eligible women who were interested in participating provided their contact information to CHWs or returned a flyer with their information to the community centre. CHWs contacted women by telephone within 1 week of initial contact, inviting them to present to the community centre to participate in a survey and biospecimen collection. Participants were not compensated for their participation in this study but were provided with a hygiene kit as an expression of gratitude for their time and participation.

### Procedures

CHWs collected 2–3 drops of capillary blood by finger prick to conduct rapid syphilis testing. Participants were given the option of providing either a ~50 cc urine specimen or self-collected vaginal swab for *Chlamydia trachomatis*, *Neisseria gonorrhoeae* and *Trichomonas vaginalis* testing. All participants opted to provide a self-collected vaginal swab. Using Xpert Swab Specimen Collection Kit (Cepheid, Sunnyvale, California), participants were instructed by CHWs to insert the swab approximately five centimetres above the vaginal opening and rotate against the vaginal wall for 30 s. The swabs were then inserted into the Xpert Swab Transport Reagent tube. The study team directly observed ~25% of all self-collection procedures and determined that participants were correctly adhering to the protocol. Specimens were immediately transported in an ice cooler to the Lebanese Ministry of Health National AIDS Control Program Laboratory in Beirut, Lebanon and kept at 4°C until analysis within 1 day of specimen collection.

Point-of-care syphilis testing was conducted using Acro Biotech rapid chromatographic immunoassays, which qualitatively detect antitreponemal IgM and IgG antibodies (Montclair, California). Vaginal specimens were tested for *C. trachomatis* and *N. gonorrhoeae* using the GeneXpert CT/NG assay, and for *T. vaginalis* using the GeneXpert TV assay (Sunnyvale, California). For each assay performed, controls were administered and automated reports were reviewed to confirm the accuracy and precision of the results. All testing data were securely and electronically stored.

CHWs were trained to administer a survey of demographic and psychosocial characteristics using validated Arabic-language tools. Demographic characteristics of interest include age, marital status, years spent in forced displacement, years spent in the current refugee camp, highest level of education attained, number of children and employment status. Psychosocial variables of interest include lifetime history of gender-based violence, which encompasses emotional, physical and sexual violence, violence in healthcare settings, war trauma exposure, post-traumatic stress disorder (PTSD), depression and food insecurity. Following the spontaneous disclosure of bothersome gynaecologic symptoms, 19 additional questions related to gynaecologic symptoms and sexual health history were added to the survey (online supplemental A). Lifetime STI history was assessed by asking participants if they had ever been diagnosed with any STI by a physician who required treatment for both themselves and their partner(s).

The NorVold Abuse Questionnaire (NORAQ) was used to screen for mild, moderate and severe forms of emotional, physical, sexual and healthcare-associated violence occurring at any time in childhood prior to age 18, in adulthood or during both periods.^31 32^ As per the NORAQ guidelines, violence severity was determined by an affirmative response to specific questions. For instance, mild emotional violence is defined as having ever experienced ‘anybody systematically and for any longer period trying to repress, degrade, or humiliate you’, while severe emotional violence is defined as ‘living in fear because somebody systematically and for a longer period has threatened you or somebody close to you’.^31^ Polyvictimisation was defined as having experienced more than one type of violence. Participants who disclosed experiencing violence were provided with psychosocial service referrals.

The number of war trauma events experienced was calculated using the ‘Trauma Events’ portion of the Harvard Trauma Questionnaire by adding the number of affirmative responses to exposure to individual traumatic acts (eg, loss of safe shelter or forced family separation), measured on a continuous scale from 0 to 40.^33^ Similarly to other studies,^34^ we defined severe war trauma exposure as having experienced greater than the median number of war trauma events reported in this sample. PTSD was defined using the ‘Trauma Symptoms’ portion of the Harvard Trauma Questionnaire, which is based on the DSM-IV R criteria for PTSD and includes 16 questions scored on a 4-point Likert scale, as a score of >2.50.^33^

Depression was measured using the WHO Well-Being Index Scale (WHO-5), which captures respondents’ agreement with five statements regarding their mood over the preceding 2 weeks, measured on a 6-point Likert scale.^35^ Participants met c theriteria for depression if they had an adjusted WHO-5 score of <28 out of 100.^36^ All participants who met cri theteria for depression or PTSD were provided referrals for mental health counselling.

Food insecurity was captured by the United Nations Food Insecurity Experiences Scale Survey Module (FIES-SM) tool.^37^ As per the FIES-SM guidelines, severe food insecurity was defined as an affirmative response to either of the following two statements, which reference any time during the preceding 12 months: ‘you were hungry but did not eat because there was not enough money or other resources for food’ and/or ‘you went without eating for a whole day because of a lack of money or other resources’.^37^

### Statistical analysis

The primary outcomes of this study were: (1) current gynaecologic symptoms, (2) self-reported lifetime STI history and (3) current STIs. Demographic, sexual history, psychosocial factors and outcome data were analysed descriptively. STI prevalence data were additionally reported as frequencies and proportions across the overall sample.

To determine, quantify and model the relationships between psychosocial factors and sexual health outcomes, we used graphical models to describe the complex interdependence structure of the variables in a compact network format. Graphical models are a popular tool for representing multivariate dependence, where the nodes of the network denote variables, and the edges (connections between nodes) represent the interdependence of the two variables based on a probabilistic model.^38^ Specifically, we applied Bayesian Gaussian copula graphical methods,^39^ developed to discover the conditional interdependence structure of variables of mixed type: binary, ordinal and continuous variables. This analytical method is ideal for examining bidirectional associations without conferring causality. Edges in the inferred graph are defined if the posterior probabilities are ≥0.5. Graphical model analysis was conducted using R V.4.2.3 (R Foundation for Statistical Computing, BDgraph package V.2.72). All other statistical analyses were conducted in Stata V.18 (College Station, Texas: StataCorp LLC).

### Patient and public involvement

Representatives of our community partner organisation, which has been operating for several decades in the refugee camp where this research was conducted, as well as the CHWs, who are refugees from the camp in which this study was conducted, were involved in the planning and implementation of this study. We initially consulted with the CHWs and our community partners to ensure that the sensitive issues raised in this study were communicated in a culturally acceptable and respectful manner. We additionally discussed the objectives and procedures of this research study prior to beginning this work. The first author continually consulted with CHWs as data collection was ongoing to review data and elicit their feedback on study procedures. Study findings were shared with CHWs and our community partner organisation at a dissemination meeting held at their community centre in the refugee camp where this study was conducted.

## RESULTS

Of the 305 eligible participants who provided their contact information, 250 participated, representing an 82.0% participation rate. Reasons for non-participation included: inability to reach participants by telephone following initial recruitment, competing responsibilities that prevented participation and refusal by the woman’s husband. No prospective participants were excluded on the basis of residence duration in the current refugee camp. Participants who had already completed the survey (n=153) were recontacted to respond to the sexual history questions added subsequently. Of these, 77.8% (n=119) completed the additional survey questions. Those who did not complete the questions had either moved away from the camp or did not respond to telephone calls or home visits by the CHW team. Our final analytic sample therefore includes demographic, psychosocial characteristics and STI testing data among 250 women aged 18–45 years (mean 30.3±SD 6.8) and additional sexual history data among a subset of 216 women. There were no significant demographic or psychosocial differences between participants lost to follow-up and the overall sample.

### Demographic, sexual history and psychosocial characteristics

**Figure 1:**
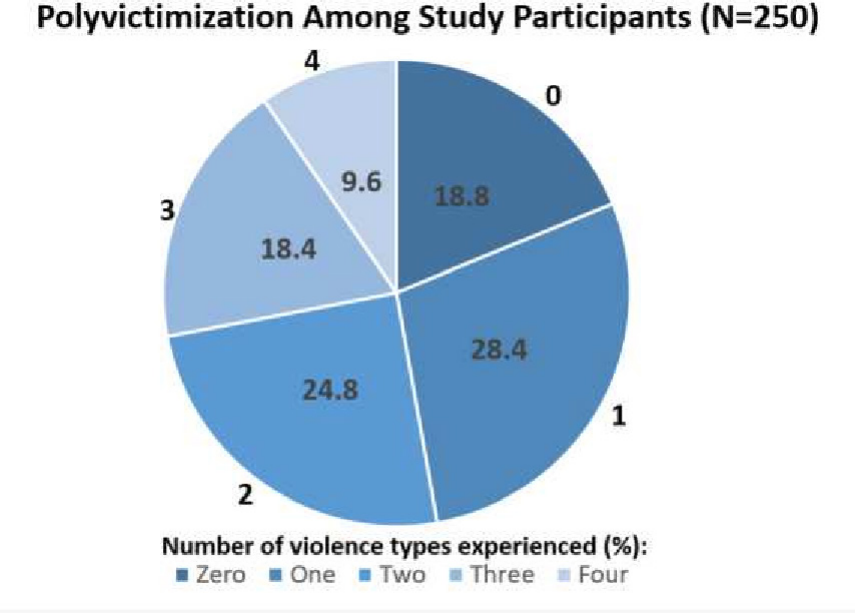
Polyvictimisation among study participants. Approximately 80% of participants experienced at least one form of sexual, physical, emotional or healthcare-associated violence. Approximately one quarter experienced two types, 18.4% experienced three types and 9.6% experienced four types of violence.

Table 1 outlines the sociodemographic, sexual history and psychosocial characteristics of participants. Over half of the participants reported living in the current refugee camp for over 5 years, indicating a protracted forced displacement setting. Participants were predominantly married and engaged in monogamous sexual partnerships. Notably, approximately 8 out of every 10 women reported experiencing sexual- and gender-based violence, including sexual (22.0%), physical (48.8%) and emotional (60.4%) violence at some point in their lifetime. An additional 8.0% (n=20) of participants experienced sexual violence as a weapon of war in Syria. Sixty-five per cent of women who disclosed experiencing violence, and 52.8% of all participants, met criteria for polyvictimisation, defined as the exposure to more than one type of violence (figure 1). Participants experienced a median of 12 traumatic war events (IQR 7) and nearly 80% met diagnostic criteria for PTSD. Over 95% of participants were food insecure and half met the criteria for severe food insecurity.

**Table 1:** Demographic, sexual history and psychosocial characteristics of participants (N=250)

	N (%)
Age, years	
18–24	58 (23.2)
25–29	54 (21.6)
30–34	60 (24.0)
35–39	52 (20.8)
>40	26 (10.4)
Marital status	
Single	1 (0.4)
Married	241 (96.4)
Divorced	6 (2.4)
Widowed	2 (0.8)
Number of children	
0	19 (7.6)
1–2	73 (29.2)
3–4	76 (30.4)
5–6	55 (22.0)
>7	27 (10.8)
Mean±SD	3.5±2.4
Mean time in Lebanon, years±SD	6.9±2.9
Mean time in current refugee camp, years±SD	5.9±3.0
Highest level of education	
Primary school or less	189 (75.6)
Some secondary school	37 (14.8)
Completed secondary school	13 (5.2)
Some university or higher	9 (3.6)
Vocational	2 (0.8)
Current employment	29 (11.6)
Mean age at sexual debut, years±SD*	18.1±3.6
<15	20 (9.3)
Mean number of lifetime sexual partners±SD*	1.1±0.2
Condom use at last sex*	23 (10.7)
Prior pregnancy	202 (93.5)
Spontaneous or induced abortion	120 (59.4)
Stillbirth	26 (12.9)
Neonatal pneumonia or conjunctivitis	14 (6.9)
Experience of violence	
Sexual	55 (22.0)
Physical	122 (48.8)
Emotional	151 (60.4)
Healthcare-associated	101 (40.4)
Any form	203 (81.2)
War trauma exposure	
1–6 traumatic events	32 (12.8)
7–12 traumatic events	103 (41.2)
13–18 traumatic events	97 (38.8)
19–24 traumatic events	18 (7.2)
Mean number of traumatic events±SD	11.9±4.6
Post-traumatic stress disorder	197 (78.8)
Depression	171 (68.4)
Food insecurity	
None	11 (4.4)
Mild	26 (10.4)
Moderate	88 (35.2)
Severe	125 (50.0)

* Data only available for 216 participants.

### Sexual health outcomes

Eighty per cent of participants reported experiencing current gynaecologic symptoms, including postcoital bleeding (24.7%), dysuria (40.3%), genital warts (13.9%), pelvic pain (41.2%), vaginal wound or ulcer (18.5%), malodorous vaginal discharge (54.9%) and green or yellow vaginal discharge (51.9%). Over half of symptomatic women were actively receiving self-prescribed treatment, including oral antibiotics (52.9%), vaginal suppositories, wash and/or ointment (56.3%), and ‘natural’ remedies (11.5%), which included vaginal rinses with boiled water and parsley, cumin, dried mint and sage, salt water, and a sodium bicarbonate solution. Nearly a quarter of symptomatic women reported using more than one type of treatment.

Sixty-three per cent of women (n=137) disclosed ever being diagnosed with an STI by a physician in their lifetime. Among these, 77.5% reported receiving treatment at the time of diagnosis. Women reporting a lifetime STI history were more likely to be on self-prescribed treatment than those without an STI history (81.6% vs 18.4%, respectively; p<0.01). Similarly, among women with a lifetime STI history, those who reported having received treatment at the time of their diagnosis were more likely to currently be on self-prescribed treatment than those who had not received treatment (93.1% vs 6.9%, respectively; p<0.001). We detected one case of *T. vaginalis*, corresponding to a prevalence of 0.004% (95% CI 0.000 to 0.020). There were no cases of *C. trachomatis*, *N. gonorrhoeae* or *T. pallidum* detected.

### Gaussian graphical model analysis

**Figure 2:**
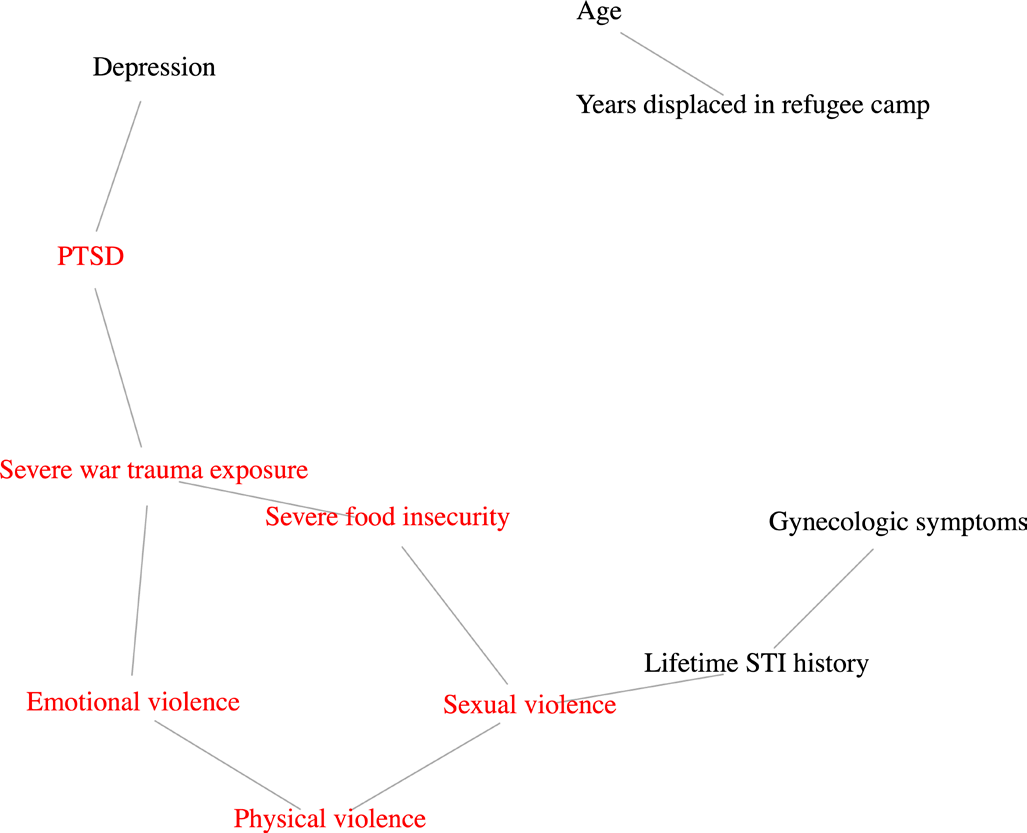
Gaussian graphical model demonstrating the syndemic clustering of psychosocial factors and health outcomes. Inferred graphical models demonstrate the clustering of six syndemic factors: sexual violence, physical violence, emotional violence, severe war trauma exposure, PTSD and severe food insecurity. Lifetime STI history is significantly associated with both gynaecologic symptoms and sexual violence. PTSD, post-traumatic stress disorder; STI, sexually transmitted infection.

Lifetime STI history was significantly associated with both current gynaecologic symptoms and sexual violence, which in turn clustered with physical violence, emotional violence, severe war trauma exposure, PTSD and severe food insecurity (figure 2). For all pairs of variables, edges are included if their posterior probabilities are more than 0.5. The posterior edge inclusion probabilities of all edges in the graph are included as a supplementary table (online supplemental B). PTSD was additionally associated with depression. Age and years spent in the current refugee camp were independently related but not associated with any psychosocial factors or sexual health outcomes.

**Figure 3:**
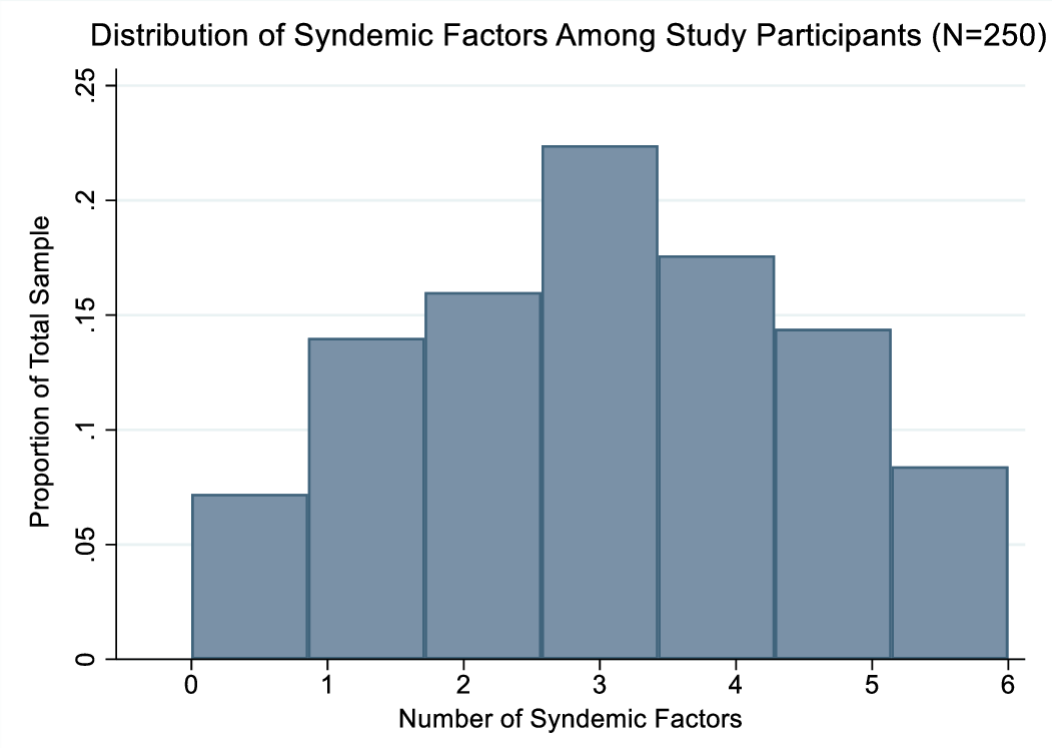
Normal distribution of six syndemic factors (sexual violence, physical violence, emotional violence, severe war trauma exposure, PTSD and severe food insecurity) represented in the Gaussian graphical model among study participants (N=250). Fourteen per cent of participants experienced one factor, 16.0% experienced two, 22.4% experienced three, 17.6% experienced four, 14.4% experienced five and 8.5% experienced all six factors. PTSD, post-traumatic stress disorder.

Approximately 93% of participants experienced at least one of the six syndemic factors (sexual violence, physical violence, emotional violence, severe war trauma exposure, PTSD and severe food insecurity) represented in the graphical model, which were normally distributed among participants (figure 3).

## DISCUSSION

To the best of our knowledge, this is the first community-based sexual health study of Syrian women who are refugees, a large and vulnerable forcibly displaced population living in a complex humanitarian setting. We determined a high prevalence of negative sexual health outcomes, including current gynaecologic symptoms and lifetime STI history. We also detected a significantly higher-than-anticipated prevalence of all-cause and sexual violence, which was strongly associated with sexual health outcomes. Our graphical model analysis indicates that forcibly displaced Syrian women may experience a syndemic of violence, war trauma, PTSD and food insecurity, which are linked with poor sexual (STI history, gynaecologic symptoms) and mental (depression) health outcomes. This novel finding, supported by our formative work in this community,^20^ suggests that women who experience one of these syndemic factors are at greater risk of experiencing others, potentially increasing the severity of the associated sexual and mental health conditions.

Over 8 in every 10 women participating in this study have experienced emotional, physical, sexual or healthcare-associated violence in their lifetime. More than half experienced multiple forms of violence. Our formative work suggests that much of the emotional, physical and sexual violence was perpetrated by intimate partners, as women in this community disclosed accounts of marital rape, physical beating, coercion and verbal abuse committed by their spouses.^20^ These figures are nearly three times greater than what has been previously reported among care-retained displaced Syrian women in Lebanon, and in the case of sexual violence, nearly seven-fold higher.^40^ While many qualitative studies suggest gender-based violence to be pervasive among Syrian women, there are few quantitative estimates, and many rely on anecdotal accounts from humanitarian organisations.^14 15^ For instance, a study among internally displaced Syrian women receiving humanitarian aid within Syria determined a 33.2% prevalence of overall intimate partner violence within the preceding 3 months, including physical (14.0%), sexual (16.8%) and emotional (24.8%) violence.^41^

The prevalence of violence detected in this study similarly exceeds that estimated in WANA, where a recent meta-analysis determined that roughly a quarter of women experienced some form of intimate partner violence in their lifetime, though studies of refugees were excluded.^42^ An older systematic review found greater variability among studies conducted in the region, in part due to the absence of a standardised screening tool and differences in sampling methods.^43^ Still, none of the included studies were among Syrian women who are refugees,^43^ reflecting a gap in the literature on violence against women in protracted displacement settings both regionally and globally, likely due to under-reporting and diminished access to health and justice systems while in displacement.^44^

There are reasons to believe that the prevalence of violence detected in this study may accurately reflect the true population prevalence among Syrian refugees in Lebanon. First, violence screening was conducted by female refugees, who are uniquely positioned to gain trust when discussing sensitive topics with women in this community. Data quality for research on violence against women may be compromised by interviewer bias, as data collectors may have adopted specific biases and stereotypes about survivors influenced by the societies in which they live.^45^ Employing displaced women from the same community as participants to conduct all data collection may have mitigated this risk. Indeed, our findings suggest that forcibly displaced women are willing to disclose personal accounts of violence to their peers in familiar settings perceived to be confidential and safe. Furthermore, our data are aligned with a growing body of literature supporting refugee-led health interventions,^46^ particularly when interventions focus on stigmatising issues.

Additionally, sexual violence data from Lebanon are relatively outdated^40^ and thus do not reflect recent compounding political, economic and humanitarian crises,^47^ which may contribute to a higher burden of violence among refugees and other vulnerable populations. Finally, though the lack of census data and sampling frames in Lebanon makes it difficult to recruit a probability-based sample, our robust community-based sampling strategy allowed for the enrolment of participants who are not regularly retained-in-care, and who would therefore have been excluded through a facility-based sampling approach. As the global refugee population is increasingly settling among host communities while being simultaneously excluded from health systems,^3^ it is imperative for researchers to develop innovative methods of recruiting this difficult-to-access population, rather than relying on clinic and other facility-based samples. Our study suggests that training refugees to implement research studies in their own communities may be an effective approach both for bolstering the representativeness of refugee study participants as well as for optimising data quality and accuracy.

Our results indicate a high burden of abnormal gynaecologic symptoms in this population, which was associated with sexual violence. Gynaecologic symptoms, including dysuria, pelvic pain, irregular menses and vaginitis, have consistently been shown to be independently and strongly associated with physical violence and sexual assault.^48 49^ Though not well understood, the relationship between violence and gynaecologic symptoms is thought to follow a ‘bio-psycho-immunologic’ model by which violence may lead to PTSD, which in turn precipitates immune modulation, rendering women more susceptible to reproductive tract infections.^50 51^ By demonstrating the clustering of both PTSD and gynaecologic symptoms with violence, our findings are consistent with this theory. Our results suggest that clinicians who encounter symptomatic Syrian women who are refugees in their practice should screen for both violence and PTSD, in addition to STIs. The integration of screening practices for violence, PTSD and STIs into routine primary and antenatal care settings may improve detection. Public health campaigns raising awareness and destigmatising these issues may also encourage disclosure among survivors. Additional alternative aetiologies to consider among our study population include bacterial dysbiosis in the setting of antimicrobial use and/or poor menstrual hygiene, contact dermatitis in the setting of using topical astringents and tissue damage due to the use of other caustic solutions reported by participants (eg, sodium bicarbonate).

Notably, we observed a discrepancy between lifetime STI history and current STI prevalence. Current STI prevalence was lower than expected for a WANA population.^52 53^ Over half of symptomatic participants were actively on self-prescribed treatment, including antibiotics, at the time of their participation in the study. Nearly a quarter were using more than one form of treatment. The unrestricted overuse of oral and topical antimicrobials may have impacted our laboratory findings through self-treatment of infections. These practices raise additional concerns about antimicrobial resistance (AMR). There is a growing literature suggesting a rise in AMR among Syrian refugees in Lebanon, where multidrug resistant organisms have been detected in refugee camp wastewater.^54 55^ A combination of care disruptions during displacement, environmental pollutants, poor sanitation and insufficient antimicrobial stewardship is thought to contribute to AMR in Syria and Lebanon,^54 56^ though AMR has not been previously examined in the context of displaced Syrian women’s sexual health. Future studies which specifically examine the prevalence of AMR among women with culture-proven STIs are needed to better understand the potential scope and magnitude of this underexplored issue. Restrictions on the dispensing of antimicrobials by pharmacists without physician prescriptions may also mitigate the risk of AMR in this population. Additionally, it is possible too that the slightly older age profile of participants in this small sample may have influenced these findings, as cervical ectopy, more common among adolescents, as well as long-term partial immunity following exposure, renders younger women more susceptible to acquiring STIs such as *C. trachomatis*.^57 58^

Alternatively, these findings may reflect a low prevalence of STIs among the general population of forcibly displaced Syrian women in Lebanon. A low curable STI prevalence in the general refugee population is aligned both with our prior research among pregnant Syrian refugee women retained in antenatal care^19^ as well as the low sexual risk behaviour profile of the sample, which is overwhelmingly engaged in monogamous partnerships. Studies among non-displaced women elsewhere in WANA demonstrate that most sexual risk behaviour is practised by men, such that women are exposed to STIs through their husbands, who may be unaware of their infections.^59^ Notably, a small number of otherwise eligible women declined study participation at the behest of their husbands; it is possible that these women may have had a higher STI prevalence, potentially mediated by their husbands. As sexual health stigma is a major barrier to men’s engagement in STI testing and treatment in this community,^20^ additional data are needed to better characterise sexual risk-behaviour and associated STI prevalence trends among Syrian refugee men. Interventional studies focused on sexual health stigma are urgently needed to better engage with both men and women in this community, particularly those experiencing intimate partner violence.

Our findings suggest the emergence of a syndemic characterised by multiple forms of violence and war trauma, which interact with other social factors, such as food insecurity, to create and perpetuate poor sexual and mental health outcomes among this vulnerable refugee population. Indeed, among Syrian women who are refugees, data indicate a greater risk of obstetric and neonatal morbidity, depression and anxiety disorders when compared with host community members,^40 60 61^ though the explicit association with social factors has not been demonstrated. A cross-sectional analysis of internally displaced Syrian women found an association between depression, emotional—but not physical or sexual—violence, and severe food insecurity, positing a potentially causal relationship with depression.^41^ Our formative work and current analysis build on this literature to suggest a bi-directional, rather than explanatory, association between violence, trauma, food insecurity and poor sexual and mental health, in the context of upstream health determinants, including limited educational, employment and social mobility opportunities among Syrian refugees in Lebanon.^20^ Put differently, this analysis is not intended to suggest a causal association between any of the identified syndemic factors. Rather, we argue that there is a bidirectional interaction, such that, for instance, women who experience sexual violence have comparatively restricted access to food and vice versa, with common root drivers (poverty, gender norms, mobility restrictions) identified in our formative work perpetuating both factors.^20 62^ Thus, a comprehensive approach which adopts the framework for syndemic effects in complex humanitarian emergencies^26^ and responds to these upstream social, political and economic health determinants is warranted to address sexual health needs and, ultimately, achieve health equity for women in this community.

There are several limitations to this study. First, there are missing sexual history data among 34 participants. However, the risk of selection bias is likely small, given no significant differences in the demographic and psychosocial characteristics of participants who were lost to follow-up. Additionally, though early marriage is common among Syrian refugees in Lebanon,^14^ we did not have ethical approval to include persons below 18 years of age in this study and were therefore unable to assess the prevalence and drivers of STIs in this vulnerable subpopulation. Furthermore, though our findings align with our qualitative data,^20^ survey data on violence and STI history are self-reported and therefore subjected to recall bias. Participants may also have inadvertently reported prior reproductive tract infection diagnoses (ie, non-sexually transmitted) as STIs. We attempted to bolster data quality by using validated scales when available and pilot-testing the survey questions, but it is possible that the true prevalence of psychosocial and clinical factors is overestimated or underestimated in this cross-sectional study. Finally, this is a study of 250 women from a single urban community in Beirut. Our findings therefore may not be generalisable to the broader population of forcibly displaced Syrian women in rural areas of Lebanon and elsewhere. Future studies among displaced women living in remote settings, as well as population-based data, are needed to assess differences in these populations. However, as the majority of the global refugee community resides in urban settings within resource-limited host countries, we believe that our findings may apply to similar protracted displacement settings, both globally and in WANA.

This study also has strengths. To our knowledge, this is the first community-based sexual health study among Syrian women who are refugees, responding to the global gap on women’s sexual health needs in protracted forced displacement settings. We implemented a multipronged community-based sampling strategy, informed by deep knowledge of and ties to the local population among CHWs and our community partner. This approach facilitated engagement with this difficult-to-access population and may have improved representativeness. Finally, we believe that the training of female refugees from the community in which this study was conducted to serve as CHWs and lead all aspects of recruitment and data collection, bolstered data quality and contributed to capacity building.

## CONCLUSIONS

Syrian women who are refugees experience a syndemic of violence, war trauma, PTSD and food insecurity, impacting their sexual and mental health. Violence against women is pervasive in this community and appears to be contributing to a high burden of gynaecologic symptoms. Negative sexual health outcomes are prevalent and are commonly managed by self-prescribed antimicrobials, which may exacerbate symptoms, compromise diagnostic investigations and increase AMR. An integrative, syndemics-informed response is urgently needed to address the multilevel socioeconomic and political determinants of health disparities and improve access to healthcare, possibly through refugee-led models of care, in this protracted forced displacement setting.

## Supplementary material

10.1136/bmjph-2025-002561 online supplemental file 1

10.1136/bmjph-2025-002561 online supplemental file 2

## Data Availability

Data are available upon reasonable request.
