# Supplementary material for: Sexual health of Syrian women in protracted forced displacement: the syndemic interplay of violence, war trauma, poor mental health and food insecurity
Source: BMJ Public Health. 2025 Jul 5;3(2):e002561. doi: 10.1136/bmjph-2025-002561 (PMC12228450; doi:10.1136/bmjph-2025-002561)
Supplement: online supplemental file 1 [file bmjph-3-2-s001.pdf]

Supplemental Material A

Sexual Health History Questions

1. Are you currently on any form of contraception? (Yes/No/Unsure)
   a. If yes, please specify: ____________________

2. Do you experience irregularities in your menstrual cycle? (Yes/No/Unsure)
   a. If yes, please specify if you experience the following:
      i. Periods occur fewer than 21 days or more than 35 days apart (Yes/No/Unsure)
      ii. Missing three or more consecutive periods (Yes/No/Unsure)
      iii. Menstrual flow that is unusually heavy or light (Yes/No/Unsure)
      iv. Duration of menstruation longer than seven days (Yes/No/Unsure)
      v. Periods accompanied by severe pain, cramping, nausea, or vomiting (Yes/No/Unsure)
      vi. Bleeding or spotting between periods (Yes/No/Unsure)
      vii. Soaking through one or more sanitary pads in one hour (Yes/No/Unsure)

3. Have you ever been pregnant? (Yes/No/Unsure)

4. Did you ever face difficulties to become pregnant? (Yes/No/Unsure)

5. Had you seen a gynecologist before getting pregnant the first time? (Yes/No/Unsure)

6. Have you ever had a pregnancy that miscarried or was aborted? (Yes/No/Unsure)
   a. If yes, how many? (Number)

7. Have you ever given birth? (Yes/No/Unsure)

8. Have you ever had a stillbirth? (Yes/No/Unsure)

9. Have you ever given birth to a baby who was diagnosed shortly after birth with an eye infection? (Yes/No/Unsure)
   a. If yes, how many? (Number)

10. Have you ever given birth to a baby who was diagnosed shortly after birth with pneumonia? (Yes/No/Unsure)
    a. If yes, how many? (Number)

11. How old were you when you had your first sexual intercourse? (Age in years)

12. What is the number of your lifetime sexual partners? (Number)

13. Have you ever been told that you have an STI? (Yes/No/Unsure)
    a. If yes, how did you contract the infection? (specify: ___________)
    b. If yes, did you receive treatment? (Yes/No/Unsure)

14. Are you currently experiencing any of the following symptoms?
    a. Abnormal vaginal discharge (Yes/No)
       i. Abnormal odor (Yes/No)
       ii. Abnormal color (Yes/No)
       iii. Abnormal texture (Yes/No)
    b. Would or ulcer in genital area (Yes/No)
    c. Pelvic pain (Yes/No)
    d. Genital warts (Yes/No)
    e. Painful urination (Yes/No)
    f. Spotting following intercourse (Yes/No)
    g. Other (specify: ____________________)

15. If you answered yes to any of the above symptoms, are you taking any treatments (including non-medicinal remedies such as herbs)? (Yes/No)
    a. If yes, please specify: _______________

16. Do you know if your husband is currently experiencing any of the following symptoms?
    a. Abnormal penile discharge (Yes/No)
       i. Abnormal odor (Yes/No)

        ii. Abnormal color (Yes/No)
        iii. Abnormal texture (Yes/No)
    b. Wound or ulcer in genital area (Yes/No)
    c. Genital Warts (Yes/No)
    d. Painful urination (Yes/No)
    e. Unusual bleeding (e.g after intercourse) (Yes/No)

17. Thinking back to the last 3 months, how often did you use a condom? (Never/Sometimes/Most of the time/Always/Unsure)

18. Thinking back to the last time you had sexual intercourse, did you use a condom? (Yes/No/Unsure)

19. In the past week, how often have you had sexual intercourse with your husband? (Number)
