# Supplementary material for: Sexual health of Syrian women in protracted forced displacement: the syndemic interplay of violence, war trauma, poor mental health and food insecurity
Source: BMJ Public Health. 2025 Jul 5;3(2):e002561. doi: 10.1136/bmjph-2025-002561 (PMC12228450; doi:10.1136/bmjph-2025-002561)
Supplement: online supplemental file 2 [file bmjph-3-2-s002.pdf]

Supplemental Material B

	Age	Years in camp	Depression	Severe food insecurity	PTSD	Sexual violence	Emotional violence	Physical violence	Lifetime STI history	Gynecologic symptoms	Severe war trauma exposure
**Age**		1	0.02	0.05	0.06	0.29	0.09	0.02	0.05	0.15	0.02
**Years in camp**	1		0.04	0.06	0.12	0.24	0.07	0.09	0.08	0.07	0.11
**Depression**	0.02	0.04		0.06	0.95	0.1	0.08	0.07	0.05	0.04	0.07
**Severe food insecurity**	0.05	0.06	0.06		0.08	0.53	0.15	0.22	0.07	0.06	0.91
**PTSD**	0.06	0.12	0.95	0.08		0.24	0.43	0.21	0.23	0.17	1
**Sexual violence**	0.29	0.24	0.1	0.53	0.24		0.46	0.99	0.53	0.1	0.28
**Emotional violence**	0.09	0.07	0.08	0.15	0.43	0.46		0.9	0.24	0.4	0.68
**Physical violence**	0.02	0.09	0.07	0.22	0.21	0.99	0.9		0.09	0.06	0.08
**Lifetime STI history**	0.05	0.08	0.05	0.07	0.23	0.53	0.24	0.09		1	0.09
**Gynecologic symptoms**	0.15	0.07	0.04	0.06	0.17	0.1	0.4	0.06	1		0.34
**Severe war trauma exposure**	0.02	0.11	0.07	0.91	1	0.28	0.68	0.08	0.09	0.34	

Supplementary Table 1. Posterior edge inclusion probabilities. The posterior edge inclusion probabilities of all possible edges across eleven variables included in graphical models are presented above. The graph reported in Figure 2 includes twelve edges for which the posterior inclusion probability is > 0.5.
